# Endogenically sourced volatiles on Charon and other Kuiper belt objects

**DOI:** 10.1038/s41467-022-31846-8

**Published:** 2022-08-09

**Authors:** Stephanie M. Menten, Michael M. Sori, Ali M. Bramson

**Affiliations:** Department of Earth, Atmospheric, and Planetary Sciences, Purdue University, West Lafayette, IN USA

**Keywords:** Volcanology, Cryospheric science

## Abstract

Kuiper belt objects (KBOs) have diverse surface compositions, and the New Horizons mission to the Pluto-Charon system allows us to test hypotheses on the origin and evolution of these KBO surfaces. Previous work proposed that Charon’s organic-rich north pole formed from radiolytically processed volatiles sourced from Pluto’s escaping atmosphere. Here, we show an endogenic source of volatiles from Charon’s interior is plausible. We calculate that cryovolcanic resurfacing released 1.29 × 10^15^–3.47 × 10^15^ kg of methane to Charon’s surface from its interior. We modeled volatile transport and found the vast majority of this volcanically released methane migrates to Charon’s poles, with deposition rates sufficient to be processed into the observed organic compounds. Irradiated methane products appear on similarly sized KBOs that do not orbit a Pluto-sized object to draw an escaping atmosphere from, so interior-sourced volatiles could be a common and important process across the Kuiper belt.

## Abstract (web summary)

We show cryovolcanic eruptions released sufficient methane to source volatile products on Charon. Irradiated methane products are found on other Kuiper belt objects, so endogenically sourced volatiles could be important across the Kuiper belt.

## Introduction

Charon, Pluto’s largest moon, was revealed by the New Horizons mission^1^ in 2015 to be a geologically diverse world whose surface features show evidence of important geologic processes. Charon preserves its geological history better than Pluto due to a lack of glaciological and aeolian processes that would otherwise erode features away. Preservation of its oldest geologic history makes Charon the ideal object to study ancient KBO processes, as many other KBOs are similar in size but have not been visited by spacecraft. Charon’s surface is characterized by two distinct geologic units, Oz Terra and Vulcan Planitia. Oz Terra preserves Charon’s original water-ice crust while Vulcan Planitia is the product of an ammonia-rich cryoflow^2,3^. Within the Oz Terra unit, a red region named Mordor Macula covers Charon’s north pole and hosts organic molecules^4,5^, thought to be processed methane. These organic products resulting from radiolytically processed methane are called tholins and can range from red to black in coloration^6^. New Horizons data suggest a similar feature exists at Charon’s south pole^4^. Charon was geologically active until anywhere from 2–4 Ga^2,7^, when its subsurface ocean froze causing widespread cryovolcanic resurfacing across the moon’s southern hemisphere^3^, recognized today as Vulcan Planitia and estimated to be around 480,000 km^2^ in surface area^8^.

Work from Grundy et al.^4^ proposed material escaping from Pluto’s atmosphere is continuously sourcing the volatiles necessary for Mordor Macula. Although this mechanism is plausible for Charon itself, Earth-based observational data has detected methane and other volatiles on the surface of many KBOs similar in size to Charon^9,10^, most of which do not orbit Pluto-sized objects with escaping atmospheres to draw volatiles from. Many of these large KBOs show evidence of surfaces that have products of irradiated volatiles, similar to Mordor Macula on Charon^6,11–13^. However, these observations often assume primordial source material of these volatiles^14,15^, and some KBOs have evidence of unprocessed methane on their surface^6^, implying a more recent source material.

**Fig. 1:**
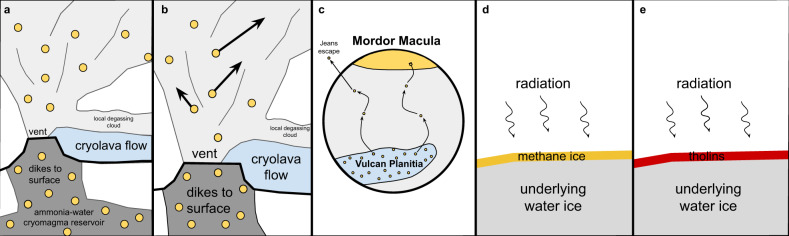
Diagram of how methane particles travel to Charon’s pole. **a**, **b** Methane (represented by yellow circles) is expelled from the subsurface through the eruption of a cryoflow onto Charon’s surface, where particles migrate across the surface with varying velocities and trajectories. **c** Some methane particles will migrate to Charon’s pole, where they become seasonally cold-trapped. Some fraction of particles will be lost from the system after reaching Charon’s escape velocity. **d** Methane ice is cold-trapped at Charon’s pole and begins to be processed. **e** Methane cold-trapped at the pole is processed over Charon’s winter to less volatile products that will not sublimate away during Charon’s summer. Over geologic time, these less volatile methane products at Charon’s pole will be processed by radiation from various sources into tholins, creating the red region we observe today as Mordor Macula.

Here, we hypothesize an endogenic source for the volatiles on these objects, including Charon, and instead propose that Charon’s widespread cryovolcanic resurfacing erupted large amounts of methane, some of which migrated to the poles and were processed into the tholins observed by New Horizons (Fig. 1). Silicate volcanism is an important process on the Earth and other rocky planets that endogenically sources volatiles to a body’s surface from its interior. Cryovolcanism could represent a similarly important process on icy bodies, including across the Kuiper belt. We test our hypothesis with a two-step process. First, we use geologic analysis to determine the total amount methane released from the emplacement of Vulcan Planitia. Second, we use a numerical model to track these volatiles as they migrated over time to determine if a sufficient amount of methane reached Charon’s poles to become cold-trapped and irradiated into the observed tholins.

## Results

### Geologic observations

**Fig. 2:**
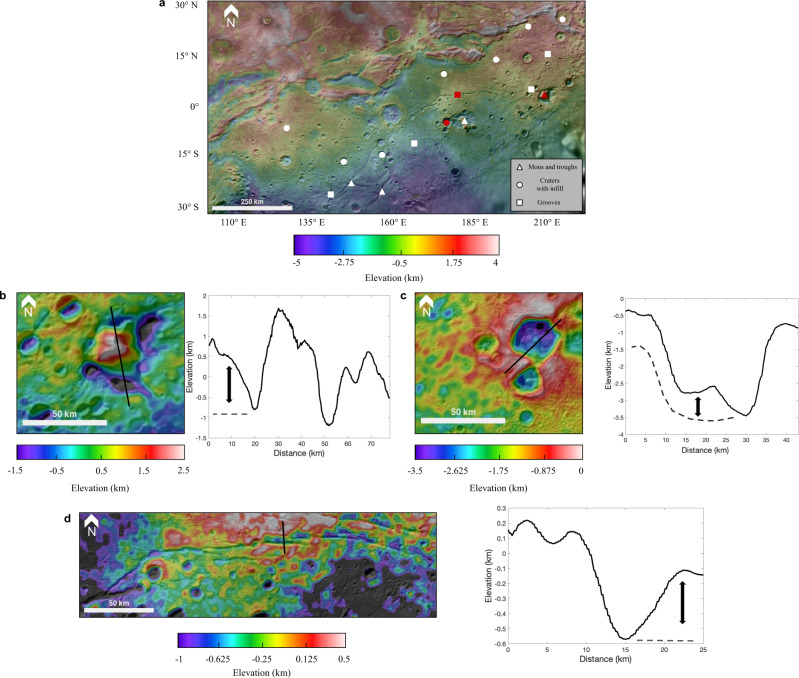
Observations of geologic features within Vulcan Planitia. **a** Locations of all geologic features that yielded Vulcan Planitia thickness constraints. Circles denote craters with infill, triangles mons and troughs, and squares tectonic grooves. **b** Kubrick Mons surrounded by a low-lying trough (red triangle in **a**) with corresponding topographic profile demonstrating measurements of the surrounding Vulcan Planitia cryoflow. **c** A crater with evidence of possible infill (red circle in **a**), and an example demonstrating the identification of thickness of the cryoflow. **d** The largest tectonic groove found within Vulcan Planitia (red square in **a**). Measurements of tectonic groove depth were taken on the side with the lowest elevation to ensure a minimum thickness constraint. All image and topography data used to generate this figure is publicly available in the NASA Planetary Data System Small Bodies node (https://pds.nasa.gov).

Geologic features observed within the Vulcan Planitia cryoflow give insight into the unit’s thickness. We used topography and imagery data^8^ from the Long-Range Reconnaissance Orbiter Imager (LORRI)^16^ on the New Horizons spacecraft to examine these geologic features in detail. We analyzed montes with surrounding troughs, craters with partial infill, and tectonic grooves (shallow fracture channels) to constrain Vulcan Planitia’s thickness at various locations across the unit (Fig. 2). Information about all the analyzed features can be found in Supplementary Table 1. These thickness values were then interpolated (see Methods) to determine the cryoflow’s average thickness, found to be around 1 km. Combining this estimated thickness with surface area estimates^8^ we found a total volume of the Vulcan Planitia cryoflow to be between 2.81 × 10^14^–7.57 × 10^14^ m^3^. This estimate is likely to be a minimum constraint, as Vulcan Planitia is thought to extend beyond the region observed by the New Horizons flyby^3,17^ and the geologic features we examined inherently give minimum thickness estimates (see Methods).

Here, the Vulcan Planitia cryoflow is assumed to primarily be an ammonia-water mixture^3^ with a small amount of other volatiles in solution. Previous literature^3^ hypothesized that the Vulcan Planitia cryoflow would be enriched with ammonia due to preferential freezing of water ice, leaving the remaining liquid ocean to be enriched in ammonia. Observation of ammonia in fresh craters within Vulcan Planitia^5^ suggests that the Vulcan Planitia cryoflow consisted of an ammonia-water mixture. Similarly, we hypothesize this flow would be saturated or close to saturation with methane due to the same freezing mechanism if any methane existed in Charon’s interior as the ocean froze. Anticipating some abundance of primordial methane on Charon is reasonable due to direct observations of methane on Pluto’s surface^18^. Pluto and Charon are hypothesized to have formed mostly from the same material^19^, so Charon should also be expected to contain some methane within its interior. Future work could consider other possible sources of methane, similar to work that has been done for the methane detected in Enceladus’ cryovolcanic plumes^20^ or if methane clathrates would be expected to form on Charon. Charon’s past subsurface ocean could have been up to 85 km below Charon’s surface^7^, creating pressure conditions in the ocean of around 250–350 bars depending on assumptions about the density of the crust. Methane solubility is 5 × 10^−3^ mole fraction in water at 300 bars of pressure^21,22^, and this value is not highly sensitive to reasonably different fractions of ammonia-water mixtures^23^. Using this fraction of methane abundance and our inferred volume constraint from geologic analysis, we found that the material that formed Vulcan Planitia would have initially contained between 1.29 × 10^15^–3.47 × 10^15^ kg of methane.

Because we likely have a conservative estimate of Vulcan Planitia’s total volume from our geological analysis techniques, our methane estimates are likely conservative as well. Vulcan Planitia is a large cryoflow that effusively resurfaced large parts of Charon with no visible evidence of any explosive volcanism, instead behaving comparable to flood basalts found on Earth. We propose viscous liquid flows erupt from various volcanic vents, consistent with hypotheses from previous work^3^, and then as the flows cool and surround features like Kubrick and Butler Mons, their viscosity will increase and flows will assume a more glacial-like behavior that is assumed from viscosity calculations in previous work^3^. We anticipate a majority of degassing occurred at source vents where viscous liquid erupts, and later changes of viscosity during emplacement will not affect this process. Flood basalts are efficient at degassing^24^, and similarly here we considered the eruption that resulted in Vulcan Planitia to efficiently degas its methane. Experimental work quantified how decreasing pressure decreases methane solubility in water^25^. We calculate that cryolavas sourced from up to 85 km below Charon’s surface would decrease in their solubility as they move from depth to the surface from 5 × 10^−3^ mole fraction to <3 × 10^−4^ mole fraction. This decrease in solubility would allow for methane gas dissolved into cryolava to be degassed efficiently from solution. Methane and carbon dioxide in terrestrial flood basalts are similarly insoluble^24^. Upon eruption, flood basalts degas their contained carbon dioxide efficiently, and geochemical analysis of mineral grains in these flood basalts reveals almost no carbon dioxide remaining (<0.002%)^26^. Methane could behave similarly in cryoflows due to its similar insolubility in ammonia-water mixtures, but future models or experimental work further quantifying this effect on Charon would be useful.

We analyzed the crater size-frequency distribution of craters within Mordor Macula to determine whether it is similar in age to Vulcan Planitia, as might be expected from our endogenic hypothesis. Mordor Macula contains a variety of craters, both those that appear to be overlaid by tholins (i.e., formed before Mordor Macula) and those that excavated both tholins and water ice material (i.e., formed after Mordor Macula). We analyzed crater densities to determine if the age of craters that appear to overlie the tholin material was significantly different than the age of Vulcan Planitia. Of the 66 total craters identified within Mordor Macula, 16 of these craters appear to be geologically younger than the tholins and have excavated water ice material onto Charon’s surface. We found that for craters in diameter greater than 20 km that excavate through tholins and into water ice, crater densities are similar to Vulcan Planitia crater densities^27^ within uncertainty (see Supplementary Figure 1). For craters with a diameter of 5 km or greater, we found a crater density of water-ice excavating craters within Mordor Macula lower than the same sized craters observed within Vulcan Planitia. However, Robbins et al. (2017) note that there is likely observational bias from both incidence angle and image resolution^27^ that obstructs our observations of craters around 5 km in size. Thus we conclude that Vulcan Planitia and the tholin layer within Mordor Macula cannot be determined to be different in age with statistical significance. The presence of craters younger than the Mordor Macula tholins implies either that the tholins formed only in the ancient past (consistent with our cryovolcanic hypothesis but not the Pluto escaping atmosphere hypothesis) or that tholin formation is sufficiently slow to not yet have resurfaced the 16 younger craters (potentially consistent with either hypothesis).

### Volatile transport modeling

We created a volatile transport model to determine the fate of methane that is erupted with Vulcan Planitia. Our model tracks the path of a methane particle as it ballistically ‘hops’ across Charon’s surface. We assume a collisionless atmosphere due to the low surface pressure and large mean free path of methane particles^4^. When a particle sublimates, the initial velocity will be based on its temperature. We used a thermal model (see Methods) to simulate Charon’s surface temperatures at different latitudes and times of the year to determine molecule velocities. Thermal inputs were then combined with ballistic transport modeling to determine a molecule’s migration path. We used a Monte Carlo approach to repeatedly track particle pathways and determine what fraction of methane released from the Vulcan Planitia cryoflow reaches Charon’s poles and what fraction escapes to space via Jeans escape (which is expected to be the dominant escape mechanism for methane on Charon^4^). We simulated 10^4^ molecules beginning at the geographic location of the Vulcan Planitia eruption and tracked their path as they migrated to the Mordor Macula region, Charon’s south pole, or until they were lost to space. Velocity of methane particles was chosen randomly from a probability distribution based on surface temperature (see Methods). Methane particles that accelerated to Charon’s escape velocity were considered to have escaped to space and removed from the simulation. Similar methods have been used to trace carbon dioxide migration on other icy moons^28,29^.

**Fig. 3:**
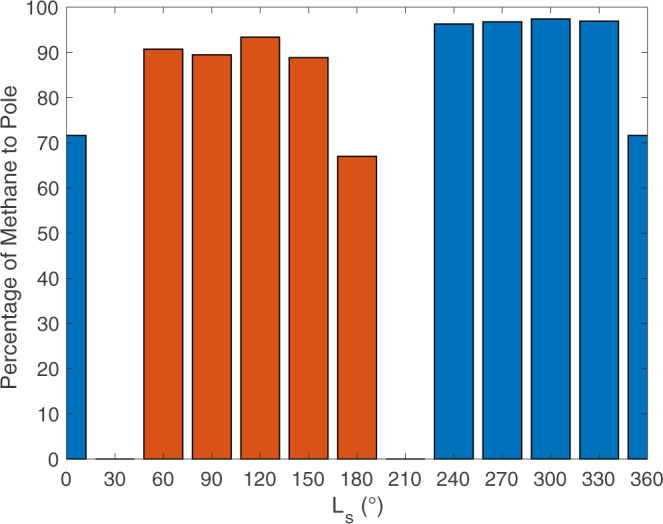
The fate of methane particles after migration as a function of solar longitude (L_s_). The percentage of methane particles that migrate to each pole over the course of one Charon year and become cold-trapped. During some times of the year at each pole (i.e., L_s_ 210–30° for Charon’s south pole and L_s_ 30–210° for Charon’s north pole), sublimation rates are high and methane ice is unstable.

We found that the vast majority of erupted methane migrates to a polar cold trap. For example, in northern winter, 97% of methane erupted from Vulcan Planitia would migrate to Charon’s north pole, and 3% escapes to space (Fig. 3). Over one Charon year, we find around 1.5–2.5 mm of methane ice would be deposited at each of the poles, assuming Vulcan Planitia was emplaced over a 1 Myr timescale. Even if the cryoflow took 1 Gyr to fully erupt and release its methane, shifting the deposition rate to around 1.5 × 10^−3^–2.5 × 10^−3^ mm per Charon year, the overall supply to the poles is still greater than the comparable exogenic rate of 0.3 μm per Charon year^4^ to source the tholins of Mordor Macula. Therefore, we find that endogenically sourced volatiles creating Mordor Macula through cryovolcanism is viable on Charon. The emplacement duration of Vulcan Planitia is uncertain. However, our estimate of the cryoflow’s total volume and methane content allows for approximation of the total amount of methane ice that migrated to Charon’s poles overall regardless of the timescale of eruption. Over the entire duration of Vulcan Planitia’s emplacement, around 9 cumulative meters of methane migrates to each pole.

#### Tholin production

During northern summer, methane ice is unstable at Mordor Macula’s latitudes and rapidly sublimates away. Southern summer produces similar rates of sublimation at Charon’s south pole. Similar to previous work^4^, we find the processing of methane ice by radiation must occur relatively quickly (over 1 Charon winter) to less volatile products that will not rapidly sublimate away during Charon summers. The most common source of radiation affecting Charon’s poles in winter are Lyman alpha photons scattered by the interplanetary medium^4^. These photons break the bonds between elements within the methane molecule and initiate the photolysis process to heavier (and less volatile) products^30,31^. Photolysis of methane ice occurs within an optical skin depth of the surface. For methane ice^4^, this optical depth is 53 nm. Grundy et al. found that in one Earth year, 1.5% of the methane within this optical depth will be photolysed. Thicknesses of methane deposition quickly reach this 53 nm optical thickness according to our range of possible methane deposition rates (our endmember for slowest deposition results in tens of nm of methane ice deposition per Earth year at the winter pole). This process of photolysis occurs continuously throughout the time when methane would be stable at each pole (~100 Earth years). Therefore, we expect ~0.1 microns of total methane ice (53 nm/Earth-year deposition × 1.5% photolysed × 100 Earth years) undergoes photolysis at each pole during that pole’s winter. This production rate is comparable to the exogenic production rate^4^ because our methane deposition rates are sufficiently high that they are not the limiting factor. These products of photolysis will not yet have reddened, because reddening occurs over kyr to Myr timescales^32^. Over geologic time, these less volatile products will continually be processed by radiation until eventually they undergo the reddening process to become tholins as we observe today. Sustained cryoflow eruptions allow for this migration and processing of methane ice to Charon’s poles to occur over an extended amount of Charon years.

Experiments demonstrate that irradiation of icy surfaces and the production of tholins is a multi-step process with different observable stages^32–34^. Irradiated surfaces move from red in color to black as they age. Mordor Macula has a red color but is likely of order Gyrs old^3,7^, so other processes are likely preventing the pole from being completely blackened. One possible explanation is a hybrid endogenic-exogenic source: that the bulk of material sourcing Mordor Macula was through the cryovolcanic mechanism proposed here, but a small amount of material is deposited at the pole through capture from Pluto’s atmosphere^4^, refreshing the very uppermost layer exposed at Charon’s surface. Impact gardening with underlying water ice through micrometeorite bombardment is another proposed explanation^4^ for preventing the blackening of Charon’s pole. Similar impact gardening could be a process that occurs in the Kuiper belt to prevent blackening of red coloration, due to the observation of red coloration on other KBOs with no Pluto-like objects to provide a continuous and recent source of material to refresh the uppermost surface layer.

## Discussion

We find that the cryovolcanism that formed Vulcan Planitia would have released a large amount of methane and other volatiles to Charon’s surface. Cryovolcanism is a potential endogenic mechanism that could source volatiles to an icy body’s surface from its interior, as for example has been proposed for Titan to replenish its atmosphere^35^. On icy objects without substantial tidal heating, large amounts of cryovolcanism may be associated with subsurface ocean freezing^36,37^. Widespread cryovolcanism preserved across icy surfaces may be common in KBOs whose subsurface oceans have frozen. KBOs similar in size to Charon or smaller (but large enough to have undergone differentiation into bodies with a rocky core surrounded by an outer ice shell and possible subsurface ocean) are all candidates for this process to endogenically source volatiles from interior to surface, including Makemake, Quaoar, Sedna, Eris, and Gonggong. KBOs of similar sizes have detectable methane or its irradiation products on their surfaces^6,10^, and these bodies often are red in color.

**Fig. 4:**
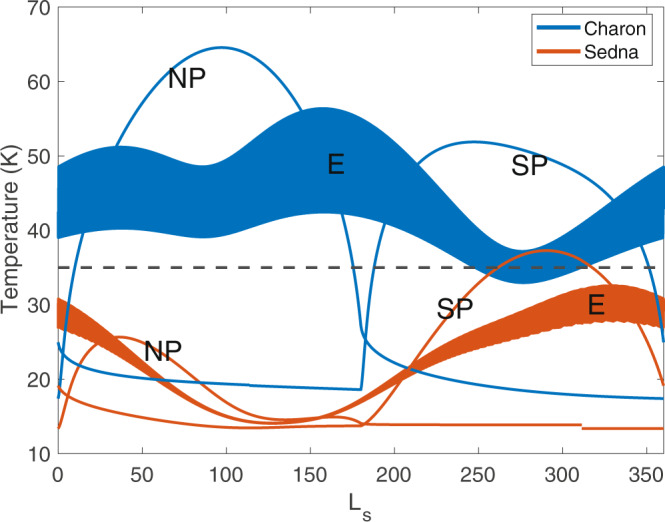
Surface temperatures of Charon and Sedna. Charon surface temperatures (blue) at the north pole (NP), south pole (SP), and equator (E) over one Charon year, and Sedna surface temperatures (orange) at the same latitudes over one Sedna year assuming a 45° obliquity. Sedna surface temperatures rarely climb above 35 K (dashed line; temperature at which cold trapping of methane is effective) at any latitude or time of year, so methane ice would be stable at all locations across Sedna’s surface for long periods throughout its orbit and would have long periods of time throughout the Sedna year to be radiolytically processed into non-volatile methane products and eventually tholins at any latitude instead of solely at the poles like Charon (see Supplementary Fig. 5).

A prediction of our endogenic hypothesis is that we expect to see evidence of radiolytically processed volatiles at the surfaces of other KBOs that do not orbit larger Pluto-sized bodies with escaping atmospheres. Makemake in particular meets this expectation of processed volatiles, where astronomical observations have demonstrated that Makemake’s surface consists of a red surface mostly made up of methane ice^6^. Previous work has proposed that unprocessed methane could be uncovered through variations in Makemake’s orbit^6^; however, methane could also potentially be sourced from Makemake’s interior through cryovolcanic eruptions as is proposed here for Charon. We used our thermal model to simulate temperatures and methane sublimation rates of objects more distant than Charon and found that some of them experience consistently low surface temperatures across all latitudes such that their entire surface acts as a “cold trap” for methane throughout their orbit (see Supplementary Figure 2). Therefore, a second prediction of our volatile transport model is that very distant objects should have volatiles or their irradiation products widely dispersed across their surfaces instead of concentrated at the poles (Fig. 4). This prediction is consistent with, for example, Sedna, where current observations suggest that the entire body is a dark red object with Earth-based detections of volatiles across the surface, including methane^13,38^. A few large KBOs do not yet have proposed evidence of extensive red, irradiated methane products on their surfaces^10^. These objects may still have active subsurface oceans, as has been proposed for Pluto^39^, that have not yet frozen and thus not yet resurfaced large portions of the body’s surface. Other large objects with low densities and albedos, such as 2003 AZ84, may not be fully condensed or differentiated^40^. Future spacecraft visiting bodies like Makemake or Sedna may observe evidence of widespread cryovolcanism across their surfaces in the form of smooth, lobate plains similar to the smooth plains of Vulcan Planitia observed on Charon, whereas objects like Eris may be found to be more Pluto-like with the possible existence of present-day oceans and no extensive volcanic plains.

## Methods

### Measurements

We interpolated the thickness of Vulcan Planitia through topographic measurements of three geologic features found within the unit: craters with partial infill, montes and their surrounding troughs, and tectonic grooves. Topographic measurements^8^ were taken using New Horizons data through the LORRI instrument^16^. Each mons, crater, or groove locally constrains thickness as outlined below.

Previous work has proposed that the montes features within Vulcan Planitia are blocks of Charon’s original crust that were surrounded by the Vulcan Planitia cryoflow^2,3^. We compared trough depth to surrounding plain heights to approximate the Vulcan Planitia thickness surrounding the main montes features, Kubrick Mons and the Clarke Montes (Fig. 2).

Because the montes features predate Vulcan Planitia, it is highly probable that some craters also existed on Charon’s surface before the emplacement of Vulcan Planitia. Small craters were almost certainly completely covered by Vulcan Planitia, but some craters were likely large enough to only be partially infilled as a result. Of the visible craters^41^ on Charon’s surface, we found that eight contain evidence of partial infill in Vulcan Planitia (Fig. 2). Landslides are a possible alternative explanation for this partial infill, but previous work on Charon’s surface^42,43^ did not identify these eight features as landslides. We measured the height of infill of each crater by analyzing topographic profiles in order to obtain an estimate of Vulcan Planitia’s thickness at that location. We used the lowest elevation in the crater as an estimation of floor depth.

Tectonic grooves were the third geologic feature used to constrain thickness (Fig. 2). These features are proposed to be tectonic in origin^8,44^, and were likely emplaced during or soon after the emplacement of Vulcan Planitia, plausibly as cooling fractures due to the cryoflow cooling. Cooling fractures on the top of flows are a common result of effusive lava flows crystallization on Earth^45^, forming in the brittle upper parts of lava flows but not penetrating to the ductile material underneath. Therefore, we considered depth measurements of the tectonic grooves to be a minimum estimate of Vulcan Planitia’s thickness at that location, as these fractures would likely not penetrate to the base of the Vulcan Planitia unit.

Our measurements of local thickness estimates range from just a few hundred meters to around two kilometers. We compared uncertainty in the topography^8^ to our inferred estimates of thicknesses, and we discarded any estimated values that were smaller than the local uncertainty in the topography data (see Supplementary Table 1). We applied a spline interpolation (see Supplementary Figure 3) to measurements and found Vulcan Planitia’s average thickness for different combinations of thickness measurements. The nominal case, which uses all thickness measurements, yielded an average thickness of Vulcan Planitia of 1 km. To guard against the possibility that our interpretations of crater infill or grooves are incorrect, we considered other cases that only used subsets of the obtained thickness measurements (e.g., from craters and grooves only, but not montes). We found that the average thickness value of Vulcan Planitia did not change substantially across permutations (it ranged from 0.58 km for the case where we considered only craters and grooves to 1.58 km for the case where we only considered montes with troughs), showing our work is robust to different geologic interpretations of individual features.

### Thermal model

Temperature inputs for ballistic transport modeling were derived from a modified thermal model^46^ using inputs specific for Charon. We used a 1D semi-implicit thermal model to determine the balance between absorbed solar energy, blackbody radiation emitted by Charon’s surface, and thermal conduction within Charon’s subsurface. This energy balance was achieved through use of the Crank-Nicolson method^47^ to numerically solve the heat equation. Thermal conduction was modeled using numerical layers, with thermal diffusion dependent on the layers’ material density (1615 kg m^−3^), specific heat capacity (837 J kg^−1^ K^−1^), and thermal conductivity (1.7 × 10^−4^ W m^−1^ K^−1^) at a specific depth. These values were based on values found for Saturnian satellites^48^, assuming an overall low thermal inertia (15 J m^−2^ K^−1^ s^−0.5^) due to high regolith porosity and the low surface pressure on airless bodies. Surface temperature values calculated are dependent on latitude, surface albedo (assumed to be 0.3), emissivity (assumed to be 0.9), time of year, longitude of perihelion (113.8°), Charon’s obliquity and eccentricity (122.53° and 0.2488, respectively), and semi-major axis (39.48 AU).

We assumed a nominal geothermal heat flux of 3 mW m^−2^ based on chondritic abundances of radiogenic heat producing elements^49^ and previous work that determined the near-surface geothermal heat flux of a Charon analog over geologic time with a ~0.64 rock fraction^50^. Our nominal value of 3 mW m^−2^ corresponds to the near-surface geothermal heat flux that would occur around potential Vulcan Planitia emplacement times, from 2–4 Ga^2,7^. However, reasonable changes in this value only minorly impact resulting temperature calculations. We modeled two endmember cases of our heat flow assumption (see Supplementary Figure 4), and found that near Charon’s equator, changes in heat flux assumptions only changes the average surface temperature by less than ~1 K. At Charon’s poles, heat flux assumptions do have a greater effect on surface temperature, but by no more than 2–3 K. This small temperature change has little effect on the cold-trapping mechanism occurring at Charon’s poles, which are always sufficiently cold for methane stability during winter.

We performed thermal modeling on Sedna to test how our hypothesized endogenic mechanism would act on KBOs much more distant than Charon. For Sedna thermal modeling, we used a semi-major axis of 506.0 AU, an eccentricity of 0.8496, and a longitude of perihelion of 311.4°. Because Sedna’s obliquity is unknown, we modeled Sedna’s surface temperature with 3 different possible obliquities, 0°, 45°, and 90° to observe the range of possible surface temperatures (see Supplementary Figure 2). Other input properties were the same as the Charon case.

### Ballistic transport model

We used a ballistic transport model to track the path of methane particles as they sublimate and ballistically ‘hop’ across Charon’s surface. For each hop, the initial velocity of a particle, *v*, was randomly picked from a half-Maxwell-Boltzmann probability distribution, *s*, described below:

1 $$s=4\pi {\left(\frac{\mu }{2\pi {k}_{b}T}\right)}^{1.5}{v}^{2}{e}^{\frac{-\mu {v}^{2}}{2{k}_{b}T}}$$

where *μ* is the mass of one methane molecule, *k*_*b*_ is the Boltzmann constant, and *T* is surface temperature in K (see Supplementary Figure 5). A methane particle’s path and distance it travels, *d*_*ball*_, is described as a function of Charon’s mass, *M*, the gravitational constant, *G*, and initial velocity, *v*, by the following equations:

2 $$a=\,\frac{1}{\frac{2}{r}\,-\,\frac{{v}^{2}}{{GM}}},$$

3 $$E=\sqrt{1-\frac{r\,\left(2a-r\right){{{\sin }}}^{2}\theta }{{a}^{2}}},$$

4 $$\omega ={{\arccos }}\left(\frac{a-a{E}^{2}-r}{{rE}}\right),$$

5 $${d}_{{ball}}=\left(2\pi -2\omega \right)r.$$

*a* is semi-major axis, *E* is eccentricity, *θ* is particle launch angle chosen randomly between 0–90° (weighted by a cos*θ* term for an isotropic distribution), and $\omega$ is the true anomaly. The escape velocity of Charon is 591 m/s. Any methane particle whose initial velocity was greater than escape velocity was assumed to escape Charon and was removed from the simulation.

We calculated sublimation rates at different latitudes throughout the Charon year using results from the thermal modeling of surface temperatures. Sublimation rate (see Supplementary Figure 6), *i*, is described as a function of vapor pressure, *p*:

6 $$i=p\sqrt{\frac{\mu }{2\pi {k}_{b}T}}.$$

The vapor pressure, *p*, of methane at a given temperature *T* is described by the Clausius-Clapeyron equation^51^:

7 $$p=11,696{e}^{10.86-\frac{985.2}{T}}.$$

At low enough temperatures, methane particles will become less likely to ballistically hop and more likely to remain trapped in place, i.e. ‘cold-trapped’. Cold-trapping of methane begins to occur most effectively around a surface temperature of 35 K and below, when sublimation rates are low.

## Supplementary information

Supplementary Information

## Data Availability

The spacecraft data come from NASA’s New Horizons mission and are also publicly available in the NASA Planetary Data System (https://pds.nasa.gov).

## Code availability

Analyses were carried out using MATLAB R2020b. Code for the volatile transport model is available at https://github.com/smenten/Charon-Cryovolcanism^52^.
