# Supplementary material for: Endogenically sourced volatiles on Charon and other Kuiper belt objects
Source: Nat Commun. 2022 Aug 9;13:4457. doi: 10.1038/s41467-022-31846-8 (PMC9363412; doi:10.1038/s41467-022-31846-8)
Supplement: Supplementary file 1 — Supplementary Information [file 41467_2022_31846_MOESM1_ESM.docx]

**Supplementary Information**

| Feature Type | Latitude | Longitude | Measured Thickness (km) | Topographic Precision (km) |
| --- | --- | --- | --- | --- |
| Crater | -15.71 | 151.34 | 0.48 | 0.40 |
| Crater | -14.03 | 162.56 | 0.61 | 0.40 |
| Crater | -6.04 | 134.52 | 0.69 | 0.40 |
| Crater | -4.52 | 181.89 | 0.94 | 0.10 |
| Crater (Discarded) | 10.08 | 180.93 | 0.27 | 0.36 |
| Crater (Discarded) | 14.15 | 197.12 | 0.34 | 0.36 |
| Crater (Discarded) | 24.38 | 206.84 | 0.28 | 0.36 |
| Crater | 26.43 | 216.42 | 0.47 | 0.40 |
| Mons + Trough | -25.69 | 162.31 | 1.50 | 0.40 |
| Mons + Trough | -22.81 | 153.88 | 1.22 | 0.40 |
| Mons + Trough  (Clarke Montes) | -3.94 | 187.25 | 1.98 | 0.10 |
| Mons + Trough  (Kubrick Mons) | 3.13 | 210.88 | 2.05 | 0.36 |
| Groove | -26.50 | 147.44 | 0.75 | 0.40 |
| Groove (Discarded) | -8.44 | 172.44 | 0.30 | 0.40 |
| Groove | -2.69 | 172.25 | 0.57 | 0.10 |
| Groove | 1.69 | 203.0 | 0.97 | 0.10 |
| Groove | 16.81 | 209.38 | 0.37 | 0.36 |

**Supplementary Table 1|** **Thickness measurements of Vulcan Planitia.** All thickness measurements made through the analysis of geologic features, including the latitude and longitude of the feature and feature type. Any inferred thickness value smaller than the precision in topography at that location was discarded.


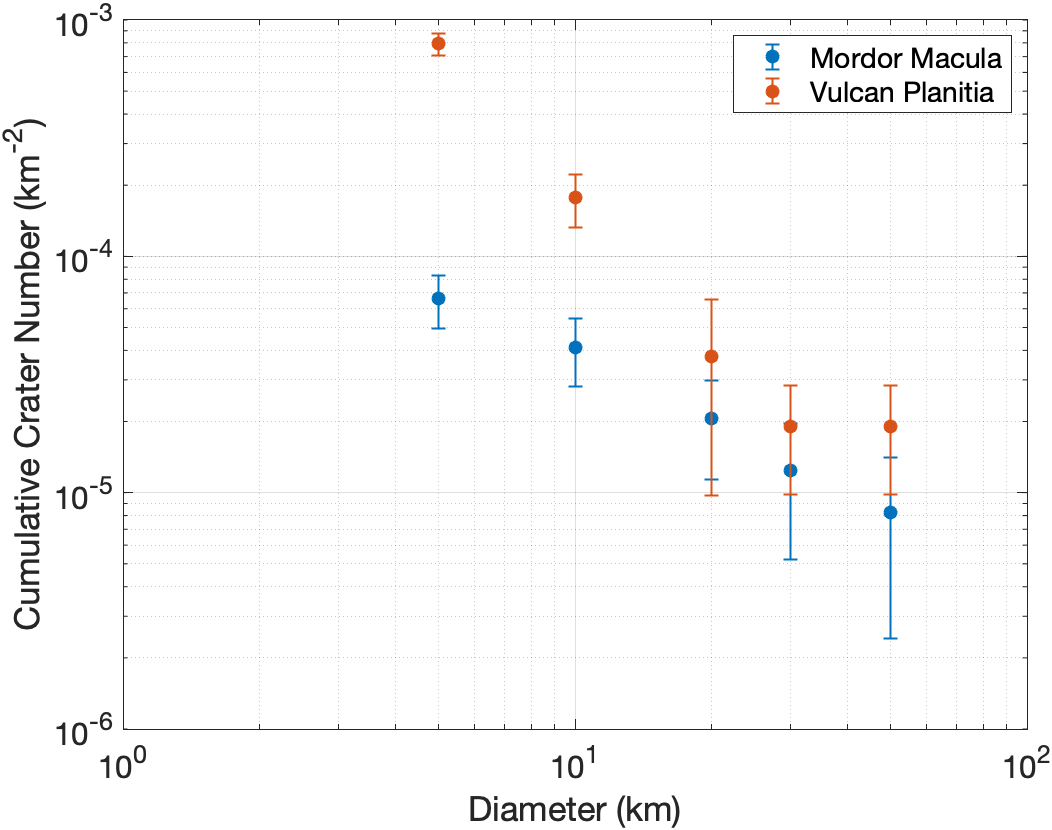


**Supplementary Figure 1| Size frequency distribution plot of Mordor Macula and Vulcan Planitia.** Crater densities of Mordor Macula for solely craters that have exposed water ice from beneath Mordor Macula’s tholin layer. We calculated densities for craters with greater than 5, 10, 20, 30, and 50 km in diameter, and compared against published crater frequency distributions^27^ of Vulcan Planitia. Crater densities for crater diameters greater than 20 km overlap in uncertainty.


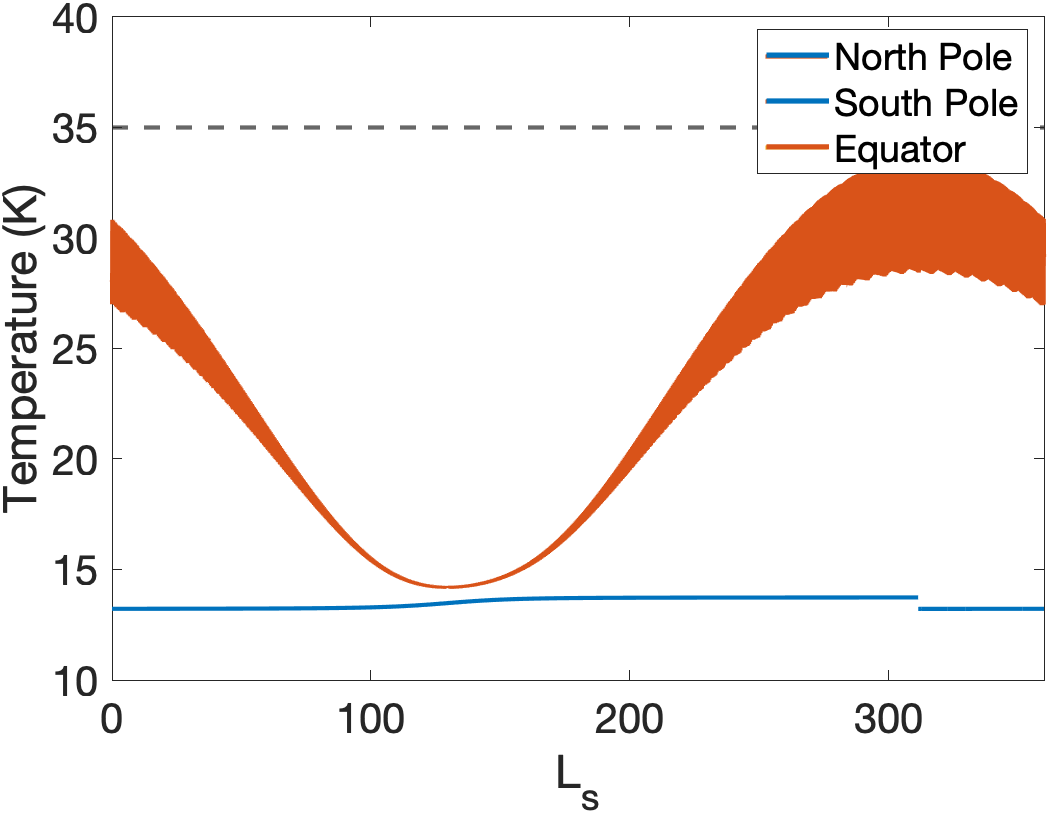

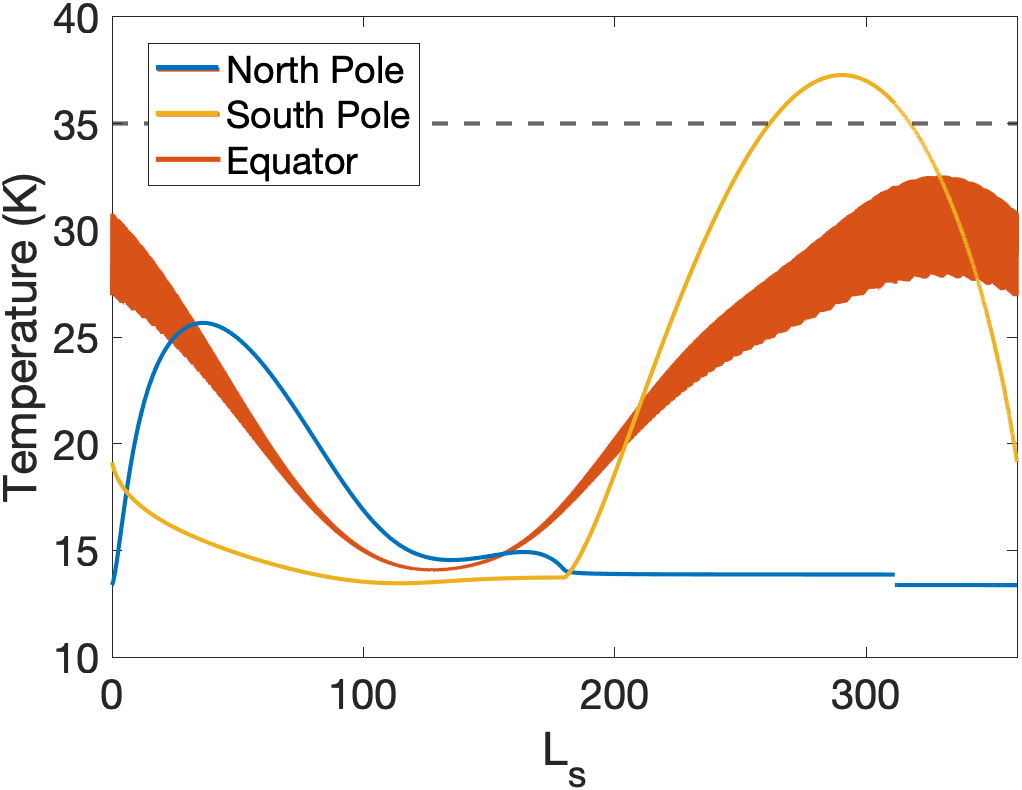

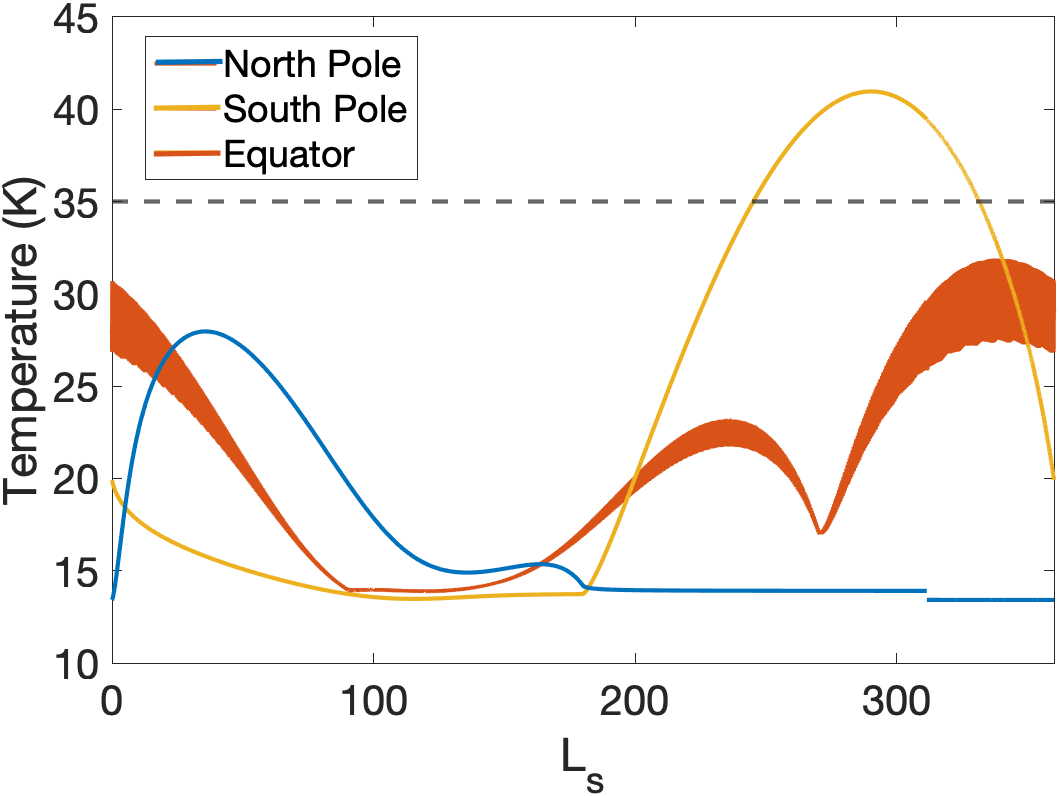


**Supplementary Figure 2|** **Sedna surface temperatures at different obliquities.** Sedna surface temperatures if the body has 0°, 45°, and 90° obliquity, respectively. At all latitudes and all ranges of possible obliquities, Sedna’s surface temperature at all latitudes remains below the temperature where methane ice is stable (35 K) for the majority of Sedna’s year.


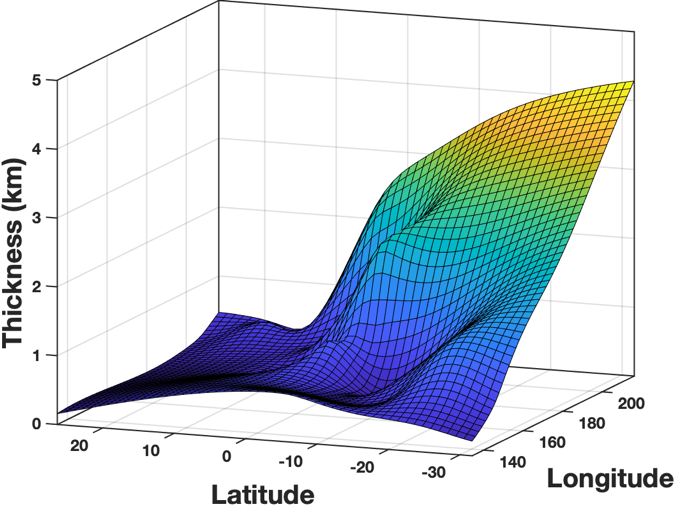


**Supplementary Figure 3| Interpolation map of Vulcan Planitia.** Interpolation of Vulcan Planitia’s thickness based on thickness constraints taken from analysis of the geologic features. Thickness ranges from only a 100 m to around 4 km, with the average thickness being 1 km across the unit.


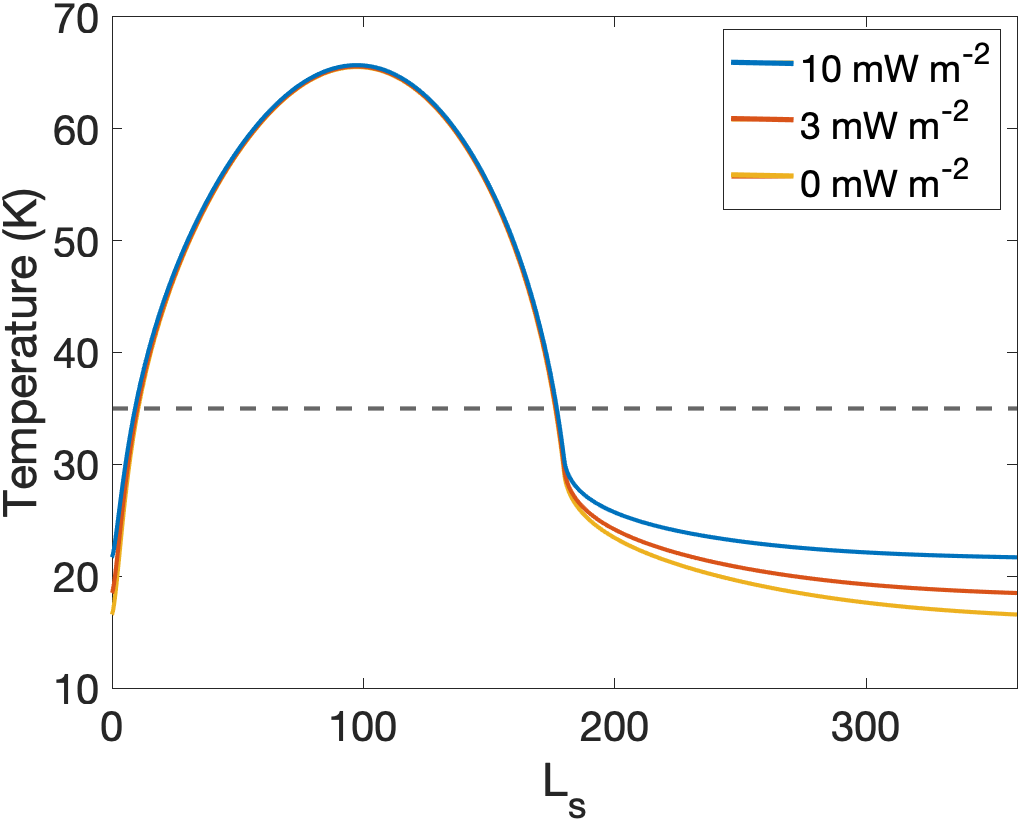


**Supplementary Figure 4| Surface temperatures with changing geothermal heat fluxes.** Models of surface temperatures at Charon’s north pole with changing geothermal heat fluxes. During the polar night, assumptions in geothermal heat flux does affect the surface temperature, but not more than 1–2 K. These changes in surface temperature models will not affect the cold-trapping mechanism present at Charon’s north pole (which begins below 35 K and is shown by the dotted line).


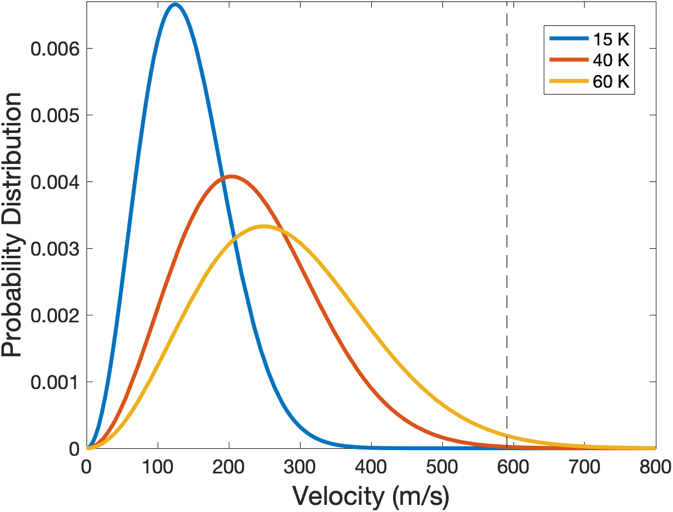


**Supplementary Figure 5| Velocity distribution of methane particles.** The probability of velocities of methane particles on Charon at different surface temperatures. Only a small quantity of particles will have a velocity above Charon’s escape velocity, and the number of particles moving above the escape velocity decreases as surface temperatures get colder.


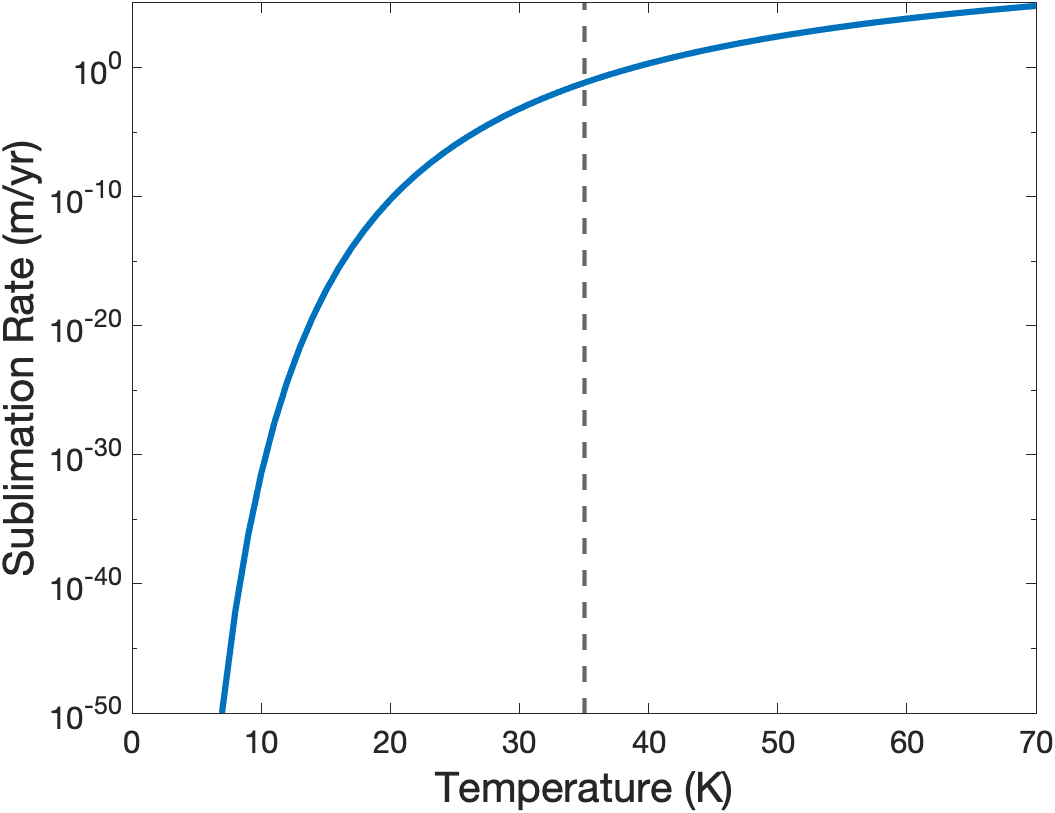


**Supplementary Figure 6|** **Sublimation rate of methane ice based on temperature.** Methane ice becomes unstable around 35 K and begins to sublimate at relevant timescales (mm/yr).
